# Characteristics of Infective Endocarditis in a Tertiary Hospital in East China

**DOI:** 10.1371/journal.pone.0166764

**Published:** 2016-11-18

**Authors:** Huimin Xu, Siyu Cai, Haibin Dai

**Affiliations:** 1 Department of Pharmacy, Second Affiliated Hospital, Zhejiang University School of Medicine, Hangzhou, Zhejiang, China; 2 Department of Cardiology, Second Affiliated Hospital, Zhejiang University School of Medicine, Hangzhou, Zhejiang, China; Virginia Commonwealth University, UNITED STATES

## Abstract

The epidemiology, clinical presentation, and treatment of infective endocarditis (IE) has significantly changed over the past few years in developed countries. However, relevant data from developing countries are different and remain scarce. The objective of this study was to evaluate the clinical presentations, treatment and outcomes of IE patients in a tertiary hospital in East China over an 8-year period. This was a retrospective observational study of consecutive cases of definite or possible IE as per the modified Duke criteria between January 2008 and December 2015. A total of 135 definite and 39 probable IE cases were identified. The mean age was 47.8 ± 15.7 years, with a male preponderance (1.9: 1). Degenerative valve disease accounted for 30.5% cases of IE, followed by congenital heart disease (29.9%) and rheumatic heart disease (14.9%). Native cardiac valves were present in 93.7% of the IE patients. Echocardiography and blood culture were performed in all patients, of whom 55.2% were found to have large vegetations (≥10 mm) and the positive rate of blood culture was 60.3%. Streptococcus remained the chief causative agent that was identified in 61.9% of culture-positive patients. Glycopeptide antibacterials and cephalosporins were the most frequently used antimicrobial drugs for IE therapy. Seventy-six (43.7%) of the IE patients were surgically treated. The mortality rate during hospital stay was 10.9%. Our data reflected clinical and microbiological profile, and treatment of IE in a tertiary hospital located in the East China.

## Introduction

Infective endocarditis (IE) is a rare but life-threatening serious disease that still has a high mortality, even in developed countries. In 2010, IE was associated with 1.58 million disability-adjusted life-years or years of healthy life lost globally [1]. A comprehensive systematic review of the literature in 21 world regions published between 1980 and 2008 revealed that almost 1 in 4 cases of IE will not survive [2]. The pattern of this disease varies worldwide [3]. The epidemiology, presentation, and treatment of IE has significantly changed over the past few years in developed countries [4–6]. There has been a reduction in rheumatic heart disease (RHD), an increase in congenital heart disease (CHD), prosthetic device implants, invasive procedures, intravenous drug users, and those with human immunodeficiency virus and diabetes mellitus, contributing to the high-risk groups [7]. The patients affected are older than in the past [8]. Clinical presentation is characterized by high rates of *Staphylococcus aureus* infection, cardiac complications, and embolic events [8]. Early surgery has become a mainstay in the treatment of IE [9]. However, relevant data from developing countries are different and remain scarce [10–13]. Few studies have been performed so far to illustrate the changes in the epidemiology, management, and outcomes of IE in China [10, 14, 15]. Thus, we evaluate the clinical presentations, treatment and outcomes of IE patients admitted to a medical center in East China over the course of 8 years.

## Material and Methods

### Study design

This study was performed at the Second Affiliated Hospital of Zhejiang University, School of medicine (SAHZU), which is a tertiary care referral hospital located in East China, with a total capacity of 2,000 licensed beds. The Departments of Cardiology and Cardiac Surgery perform 12,000 echocardiograms, 13,000 cardiac procedures and surgeries every year, with 176,000 outpatient visits and 9,000 admissions/year. The hospital charts of patients admitted from January 2008 and December 2015 to the SAHZU with a clinical diagnosis of IE were retrospectively reviewed by two of the authors (H.X. and S.C.) independently. Patients with definite or possible IE according to the modified Duke criteria [16, 17], were included in the study. Disagreements were resolved by discussion. This study was approved by the ethics committee of the 2^nd^ affiliated hospital, school of medicine, Zhejiang university. Due to the retrospective nature of the study, informed consent was waived.

### Data extraction

For each IE patient, these two researchers independently extracted data into a pre-designed data collection form. The data included patients’ age, sex, duration of illness before hospital admission, previous antibiotic use (within 2 weeks before admission), history of recent medical procedures (within 6 months before admission) or intravenous drug abuse, co-existing disease (diabetes and chronic hemodialysis), predisposing heart diseases (CHD, RHD, or degenerative heart disease), echocardiographic findings, microbiologic data, pharmacological and surgical treatment, pathologic findings, complications (neurological events, systemic embolism, and congestive heart failure), and in-hospital mortality (dead or moribund before discharge). A neurological event was defined as symptomatic or asymptomatic stroke, hemorrhage, or encephalopathy. Systemic embolization was defined as an embolic event outside of the central nervous system. All blood cultures used to evaluate IE were obtained and processed by the microbiology laboratory of the SAHZU by standard methods to identify bacterial and fungal species and antimicrobial susceptibility profiles. These data were then analyzed generally or individually at 4-year intervals over the course of 8 years.

### Statistical analysis

Statistical analysis was conducted using the SPSS 20.0 for Windows. Descriptive statistics were calculated, including means and standard deviations for continuous variables, and frequencies for qualitative variables. The *chi-square* or *Fisher’s* tests were used to compare qualitative variables, and *Student’s t*-test was used to compare quantitative variables. The standard level of significance, *P*<0.05 was chosen. Data were shown as mean ± SD or as number of patients and percentage.

## Results

### Baseline characteristics

During this 8-year study period, a total of 183 consecutive patients with a clinical diagnosis of IE were identified. Nine patients were excluded because they did not meet the modified Duke criteria. Thus, 174 patients presented with 135 definite and 39 probable IE defined according to the modified Duke clinical criteria were included in the study. More patients had a “definite IE” diagnosis based on pathological criteria during 2012–2015, compared with the patients diagnosed during 2008–2011 (46.3% *vs*. 28.8%, *P* = 0.022) (Table 1). Of all patients included in this study, 115 (66.1%) were male and 59 (33.9%) were female, with a mean age of 47.8 ± 15.7 years (interquartile range, 36.0–61.2 years). One hundred and five (60.3%) of 174 patients were admitted to the hospital within the first month of showing symptoms of IE, and 130 (74.7%) patients had received a previous course of antibiotic therapy (Table 2).

**Table 1:** Comparison of diagnostic criteria for infective endocarditis in two time periods.

Criteria	2008–2011	2012–2015	*P* value
Total	66	108	
Definite IE	48 (72.7)	87 (80.6)	0.230
Pathological criteria			
Histology	19 (28.8)	50 (46.3)	0.022*
Clinical criteria			
2 major criteria	14 (21.2)	29 (26.9)	0.403
1 major + 3 minor criteria	15 (22.7)	8 (7.4)	0.004*
Possible IE	18 (27.3)	21 (19.4)	0.230
1 major + 1 minor criteria	17 (25.8)	21 (19.4)	0.328
3 minor criteria	1 (1.5)	0	0.379

Data are presented as number of patients (%).

*P < 0.05 was considered statistically significant.

IE, infective endocarditis.

**Table 2:** Clinical characteristics of 174 infective endocarditis patients.

Variable	2008–2011	2012–2015	*P* value
Male	40(60.6)	75(69.4)	0.232
Mean age (yr)	46.3 ± 16.1	48.7 ± 15.5	0.536
Symptom to admission <1 mo	36 (54.5)	69 (63.9)	0.222
Previous antibiotic use^a^	50 (75.8)	80 (74.1)	0.804
Recent medical procedures^b^	5 (7.6)	8 (7.4)	1.000
Diabetes mellitus	5 (7.6)	6 (5.6)	0.833
Chronic hemodialysis	1 (1.5)	5 (4.6)	0.410
Predisposing heart disease			
CHD	23 (34.8)	29 (26.9)	0.307
RHD	13 (19.7)	13 (12.0)	0.169
Degenerative	17 (25.8)	36 (33.3)	0.314
Native valves	63 (95.5)	100 (92.6)	0.666
Affected valves			
Aortic	27 (40.9)	48 (44.4)	0.648
Mitral	38 (57.6)	64 (59.3)	0.827
Tricuspid	6 (9.1)	12 (11.1)	0.671
Pulmonary	8 (12.1)	5 (4.6)	0.127
Other	2 (3.0)	9 (8.3)	0.283
Severe valve regurgitation	37 (56.1)	64 (59.3)	0.678
Severe valve stenosis	1 (1.5)	7 (6.5)	0.262
Left-sided IE	56 (84.8)	96 (88.9)	0.436
Right-sided IE	13 (19.7)	21 (19.4)	0.967
Vegetation			
Size of ≥10 mm	26 (39.4)	70 (64.8)	0.001*
Size of ≥20 mm	10 (15.2)	17 (15.7)	0.917
Mobile vegetation	18 (27.3)	60 (55.6)	<0.001*
Culture negative	27 (40.9)	42 (38.9)	0.792
Surgery	21 (31.8)	55 (50.9)	0.014*
Perivalvular abscess	1 (1.5)	7(6.5)	0.262
Perforation of the leaflet	8 (12.1)	16 (14.8)	0.617
Chordal rupture	4 (6.1)	8 (7.4)	0.975
Complications			
Stroke	16 (24.2)	19 (17.6)	0.288
Embolism, nonstroke	11 (16.7)	15 (13.9)	0.618
Congestive heart failure	45 (68.2)	75 (69.4)	0.861
Dead or moribund	8 (12.1)	11 (10.2)	0.691

CHD, congenital heart disease; RHD, rheumatic heart disease.

^a^ means within 2 weeks before admission;

^b^ means within 6 months before admission.

*P < 0.05 was considered statistically significant.

### Underlying disease

Recent medical procedures were identified in 13 patients (7.5%). Only 1 patient had a history of intravenous drug abuse. There were 11 patients (6.3%) who had diabetes and 6 patients (3.4%) who had chronic hemodialysis. A predisposing heart disease was found in 128 (73.6%) of the patients. Underlying heart disease included: degenerative valve disease in 53 (30.5%) patients, CHD in 52 (29.9%) patients, and RHD in 26 (14.9%) patients. Native cardiac valves were present in 163 (93.7%) of the IE patients (Table 2). Nine (5.2%) patients had prosthetic valve IE and 2 (1.1%) patients experienced pacemaker lead IE.

### Echocardiographic features

All patients had undergone transthoracic echocardiography, of whom 96 (55.2%) were found to have large vegetation (≥10 mm). The vegetation was 20 mm or more in size in 27 (15.5%) patients. Mobile vegetation was observed in 78 (44.8%) patients. More patients were found to have vegetation size of ≥10 mm and mobile vegetation during 2012–2015, compared with the patients diagnosed during 2008–2011 (*P*<0.05). Affected valves were aortic valve in 75 (43.1%) patients, mitral valve in 102 (58.6%) patients, tricuspid valve in 18 (10.3%) patients, and pulmonary valve in 13 (7.5%) patients. One hundred and fifty-two patients (87.4%) had left-sided IE, and 34 (19.5%) patients had right-sided IE. Severe valve regurgitation was observed in 101 (58.0%) patients, and severe valve stenosis was diagnosed in 8 (4.6%) patients. Echocardiographic characteristics are depicted in Table 2.

### Microbiological data

Blood cultures were performed in all patients. The positive rate was 60.3% (105 patients). There was no difference in positive culture rate between patients with or without prior antibiotic use before admission (62.3% and 54.5%, respectively; *P* = 0.363), and between 2008–2011 with 2012–2015 (59.1% and 61.1%, respectively; *P* = 0.792). Streptococcus remained the chief causative agent of IE (65 of 105 patients, or 61.9%), of which viridians group streptococci was identified in 61 patients, and other streptococci in 4. Staphylococci were identified in 24 of the 105 patients (22.8%): of which *Staphylococcus aureus* was identified in 11 patients, coagulase negative staphylococci in 13. Methicillin-resistant *staphylococcus aureu* (MRSA) and methicillin-resistant coagulase-negative staphylococci (MRCNS) accounted for 57.9% and 61.5% of *staphylococcus aureus* and coagulase-negative staphylococci, respectively. *Enterococcus faecalis* was identified in 5 patients. Other pathogens identified by blood cultures were: *Rothia dentocariosa* in 1 patient, *Haemophilus parainfluenzae* in 1, *Leuconostoc lactis* in 1, *Prevotella Shan and Collins* in 1, *Pseudomonas aeruginosa* in 1, *Klebsiella pneumoniae* in 1, *Acinetobacter baumannii* in 1, and *Candida* in 2 patients. Polymicrobial infection was identified in 2 patients. Microbiological data in 105 patients with positive blood cultures are depicted in Table 3.

**Table 3:** Microbiologic etiology in 105 patients with positive blood cultures.

Pathogen of endocarditis	2008–2011	2012–2015	*P* value
Streptococci	22 (56.4)	43 (65.2)	0.373
Viridans group streptococci	22	39	
Other streptococci	0	4	
Staphylococci	10 (25.6)	14 (21.2)	0.602
*Staphylococcus aureus*	6	5	
Coagulase-negative staplylococcus	4	9	
*Enterococcus faecalis*	1 (2.6)	4 (6.1)	0.649
*Candida*	1 (2.6)	1 (1.5)	1.000
Other bacteria	3 (7.7)	4 (6.1)	0.709
Polymicrobial	2 (1.9)	0	0.136

### Treatment

All patients received antimicrobial therapy. Antimicrobial regimens included: glycopeptide antibacterial in 104 patients (59.8%), cephalosporin in 87 patients (50.0%), linezolid in 40 patients (23.0%), benzylpenicillin in 40 patients (23.0%), beta-lactam/beta-lactamase-inhibitor combinations in 37 patients (21.3%), fluoroquinolones in 36 patients (20.7%), aminoglycosides in 23 patients (13.2%), carbapenems in 21 patients (12.1%), daptomycin in 7 patients (4.0%), clindamycin in 6 patients (3.4%), fusidic acid in 5 patients (2.9%), macrolides in 4 patients (2.3%), antifungals in 2 patients (1.1%), and rifampicin in 1 patients (0.6%). More daptomycin, and less beta-lactam/beta-lactamase-inhibitor combinations, aminoglycosides, fluoroquinolones, and macrolides were used during 2012–2015, compared with the drug regimens during 2008–2011 (Table 4).

**Table 4:** Anti-infectives for the treatment of infective endocarditis.

Medications	2008–2011	2012–2015	*P* value
Glycopeptide antibacterials	40 (60.6)	64 (59.3)	0.860
Linezolid	10 (15.2)	30 (27.8)	0.055
Daptomycin	0	7 (6.5)	0.045*
Fusidic acid	3 (4.5)	2 (1.9)	0.369
Benzylpenicillin	12 (18.2)	28 (25.9)	0.239
Cephalosporins	28 (42.4)	59 (54.6)	0.118
Beta-lactam/beta-lactamase-inhibitor combinations	20 (30.3)	17 (15.7)	0.023*
Carbapenems	8 (12.1)	13 (12.0)	0.987
Aminoglycosides	18 (27.3)	5 (4.6)	<0.001*
Fluoroquinolones	19 (28.8)	17 (15.7)	0.039*
Macrolides	4 (6.1)	0	0.020*
Clindamycin	1(1.5)	5 (4.6)	0.410
Rifampicin	1 (1.5)	0	0.379
Antifungals	1 (1.5)	1 (0.9)	1.000

*P < 0.05 was considered statistically significant.

Seventy-six (43.7%) IE patients were surgically treated. The percentage of patients undergoing surgery was much higher in the recent 4 years (Table 2). Forty-four IE patients underwent valve replacement, 6 underwent valvuloplasty, 1 underwent pacemaker lead extraction, 8 underwent CHD repair, 13 underwent valve replacement and valvuloplasty, 2 underwent valvuloplasty and CHD repair, 1 underwent pacemaker lead extraction and CHD repair, and 1 underwent valve replacement and CHD repair.

### Outcome

Clinical complications during hospital stay included: neurological events in 35 (20.1%) cases; systemic embolism in 26 (14.9%) cases, and congestive heart failure in 120 (69.0%) cases. During the surgery, 8 (4.6%) patients were found to have perivalvular abscess, 24 (13.8%) were found to have perforation of the leaflet, and 12 (6.9%) were found to have chordal rupture. The mortality rate during hospital stay was 10.9% (Table 2).

## Discussion

In this study, 174 IE cases were reviewed. The mean age of patients with IE were 47.8 ± 15.7 years, which was similar to the mean age of IE patients reported from Turkey (45.2 y) [18], but younger than the average age reported from the developed countries, such as Europe (61.4 y) [8]. Compared with the mean age reported from another study conducted in China from 1998 through 2009 [10] and other developing countries [11, 12, 19], the mean age of IE patients was older. Although there was no statistically significant difference in patients’ mean age between the two consecutive periods in our study, a shift toward an increased mean age was observed, which might relate to the decrease of RHD and CHD, and to the increase of degenerative valve disease.

Echocardiography is very helpful in establishing the diagnosis of IE and remains the main accurate imaging modality to identify endocardial lesions associated with IE [4]. In this study, all patients had had transthoracic echocardiography. The most commonly affected valve was the mitral valve, followed by the aortic valve and then the tricuspid valve. A trend of less pulmonary valve affected was observed during two consecutive periods, although not significantly. Interestingly, the percentage of patients with vegetation size of ≥10 mm and mobile vegetation was significantly higher in the recent four years, which might be attributed to the technical advances in echocardiography.

Blood culture remains a cornerstone of diagnosis for IE [5]. In some developed countries, the rate of negative blood cultures was low (7%~20%), and streptococci had fallen into the second place after staphylococci as the leading pathogen causing IE [7, 8]. However, in our study, the rate of negative blood cultures was 39.7%, and streptococcus was still the chief causative pathogen (61.9% of the patients with positive blood cultures). Since there was no difference in positive culture rate between patients with or without prior antibiotic use before admission, the high rate of negative blood cultures might not have resulted from the high rate (74.7%) of prior antibiotic use before admission. Chronic hemodialysis, diabetes, and intravascular devices are considered as the three main factors associated with IE due to *Staphylococcus aureus* [20]. The relative lower frequency of staphylococcus infection in our study might be due to that only 6.3% patients had diabetes, 3.4% patients had chronic hemodialysis, 5.2% patients had prosthetic valve, and 1.1% patients had pacemaker. The high frequency of streptococcal infection probably in part reflects poor dental health in Chinese patients, suggesting the need to promote oral hygiene [6, 21].

Prompt antibiotic therapy can avoid the incidence of severe sepsis, multiple organ dysfunction syndrome, risk of stroke, and sudden death. Therefore, appropriate antibiotic therapy should be initiated as soon as possible after microbiological test in suspected or confirmed IE cases [4]. In this study, all patients received antimicrobial therapy. Since streptococci remained the chief causative agent of IE and are usually susceptible to benzylpenicillin, the most frequently used antimicrobial drugs would be benzylpenicillin or cephalosporins. Unexpectedly, we observed that glycopeptide antibacterials were the most frequently prescribed antimicrobial drugs, which might be the consequence of the low positive rate of blood culture. In cases of negative cultures, antimicrobial therapy needs to cover all likely pathogens [12].

In addition to appropriate antibiotic therapy, surgery is crucial for optimal treatment in selected patients with complicated IE [6, 22]. During the past decade, valve replacement rates for IE steadily increased in developed countries [7]. The 2009 European guidelines provide clear recommendations on the indications for surgery during the active phase of the disease [23]. In the present study, 43.7% of the patients underwent surgery, which is similar to the data reported in the International Collaboration on Endocarditis-Prospective Cohort Study [8]. The percentage of patients undergoing surgery was much higher during 2012–2015, compared with the patients admitted during 2008–2011 (50.9% *vs*. 31.8%, *P*<0.05), which might be partly related to the increased number of patients with vegetation size of ≥10 mm and mobile vegetation in echocardiographic findings.

Despite advances in pharmaceutical and surgical treatment, IE remains a fatal disease [22]. The in-hospital mortality for IE has not improved, and stroke, embolism, other than stroke, heart failure and other complications remain common [8]. The in-hospital mortality rate in our study was 10.9%, which is similar to the reports from the developed countries [8, 24], but lower than the reports from Spain [25] and India [12]. There was no significant difference in mortality between the two consecutive periods in our study. The most common complication seen in 69.0% of patients in our study was congestive heart failure, which was mainly because the high rate of valve regurgitation (58.0% of the IE patients). There were 35 (20.1%) patients had neurological events, and 26 (14.9%) cases had systemic embolism.

It should be pointed out that our study is based on retrospective observation and analysis of the data collected in our hospital, which is located in the east area of China. We cannot exclude geographic as well as economic differences in comparison with other parts of China. Long-term mortality rate was not available and the sample size was relatively small. Despite these limitations, this study provides valuable data on the clinical characteristics of IE in developing countries.

Thus, our data reflect clinical and microbiological profile, and treatment of IE in a tertiary hospital located in the east of China. Our study has revealed that IE patients in our hospital are older, have lower incidence of RHD and CHD, higher rate of surgery, and lower incidence of in-hospital death, in contrast to other reports from developing countries [11, 12]. However, the mean age of IE patients is younger as compared with that in the West. Moreover, viridians group streptococci remain the leading causative pathogen of IE, reflecting the persistent poor dental health in China, while blood culture positive rate is still low. More population-based, and multicenter hospital-based studies are needed to learn more complete knowledge of IE in China.
